# Estimates of global research productivity in using nicotine replacement therapy for tobacco cessation: a bibliometric study

**DOI:** 10.1186/s12992-018-0335-z

**Published:** 2018-01-30

**Authors:** Sa’ed H. Zyoud

**Affiliations:** 1 Poison Control and Drug Information Center (PCDIC), College of Medicine and Health Sciences, An-Najah National University, Nablus, 44839 Palestine; 2 Department of Clinical and Community Pharmacy, College of Medicine and Health Sciences, An-Najah National University, Nablus, 44839 Palestine

**Keywords:** Bibliometric, Tobacco smoking, Tobacco cessation, Scopus, Nicotine replacement therapy, NRT

## Abstract

**Background:**

Tobacco use is a major healthcare problem worldwide. Tobacco smoking remains the most important risk factor for both cancer and heart diseases. This study was initiated due to the lack of published data concerning the real progress in research output in the use of nicotine replacement therapy (NRT) for tobacco cessation. This study was aimed to use bibliometric analysis to estimate the NRT literature indexed in Scopus database at global level.

**Methods:**

Core of the search strategy was the documents that contained specific words or phrases regarding NRT as keywords in the title. Publication output of most prolific countries was adjusted to the gross domestic product and population size. All citations analysis were accomplished on December 22, 2017.

**Results:**

A total of 2138 references were retrieved and published from 56 countries, which were published between 1970 and 2016. The USA has the most number of published articles accounted to 986, followed by the UK (312 publications) and then Australia (102 publications), and Sweden (102 publications). No data related to NRT were published from 156 countries. No significant correlation was found between the country population size or 2016 gross domestic product values and the number of publications of the top-10 most prolific countries in the field of NRT (*r* = − 0.156, *P* = 0.664; and *r* = − 0.173, *P* = 0.632, respectively). Furthermore, there is no correlation between prevalence of tobacco smoking and number of publications of the top-10 most prolific countries in the field of NRT (*r* = − 0.235, *P* = 0.514).

**Conclusions:**

The present data reveal a solid mass of research activity on NRT. The USA was by far the predominant country in the amount of NRT-based research activity. NRT-based research activities were low or not available in most countries. The results of this study delineate a framework for better understanding the situations of current NRT research and prospective directions of the research in this field which could be applied for managing and prioritizing future research efforts in NRT research.

## Background

Tobacco use is a major healthcare problem worldwide. Tobacco smoking remains the most important risk factor for both cancer and heart diseases causing about 6 million deaths and hundreds of billions of dollars burdens at global level each year [1, 2]. The World Health Organisation Framework Convention on Tobacco Control (WHO FCTC) recommends that governments institute broad national tobacco control policies, which should contain the provision of treatment for tobacco dependence, including use of effective smoking-cessation strategies and counselling services [3]. Medication therapy such as nicotine has been shown to help in smoking cessation [4]. This type of medication is called Nicotine Replacement Therapy (NRT) [4]. NRT has been widely accepted as the first-line pharmacological intervention for tobacco dependence because of its safety and efficacy profile [5–7].

Since the development of NRT in 1978, tobacco cessation options have continued to evolve and expand [8]. The aim of NRT is to reduce or prevent withdrawal symptoms related to quitting smoking by substituting the nicotine from tobacco smoking to reach complete abstinence from tobacco smoking [9–12]. NRT is available in various forms, e.g. nicotine skin patches, chewing nicotine gum, lozenges/tablets, nasal and oral sprays and inhalers [6, 7]. All forms of NRT were all significantly more effective than placebo, or no NRT, as part of a strategy to promote smoking cessation [6].

Tobacco smoking is on the rise, and since smoking behaviours and consequences involve a multidisciplinary research approach, there is an increasing level of research that includes almost all of worldwide regions that have interested in health sciences production [13, 14]. Research output has an important function in the scientific development providing a key association between knowledge generation, and use [15]. Although, to the author’s knowledge, there were small number of bibliometric studies in the field of tobacco smoking were conducted [2, 14, 16–20], no bibliometric studies have been conducted in the field of NRT. Bibliometric is the application of quantitative analysis method based on statistics and mathematics within a given certain topic [21–23]. This study was aimed to use bibliometric analysis to estimate the NRT literature indexed in *Scopus* database at global level, and to identify hotspots in research related to NRT. Results from this study will allow researchers to identify the hotspots in NRT which may open doors to new research on the development of effective smoking-cessation strategies and counselling services. Additionally, results delineate a framework for a perceptive recognition of current NRT research and as a result of a realistic advice for decision makers.

## Methods

### Search strategy

The analysis of the scientific research output in this bibliometric study was based on previous bibliometric studies [2, 14, 24–27]. *Scopus* database was used as the source to retrieve all the bibliometric data regarding the research output in the field of NRT. The *Scopus* database was used because it is the largest citation and abstract database of peer-reviewed Journals, and it is one of the most reliable databases for publications and citations. Moreover, this database has been widely used in several bibliometric studies in the field of tobacco use [2, 14, 19, 28]. Furthermore, *Scopus* database has been commonly employed in several bibliometric analyses due to combining in the characteristics of both Web of Science and PubMed [29–34].

Documents were selected including the keywords related to NRT that were chosen from previous studies may have been reviews, practical reporting or meta-analyses [6, 35–41]. Keywords used are: ‘nicotine replacement’, ‘NRT’, ‘nicotine patches’, ‘nicotine gum’, ‘nicotine transdermal’, ‘nicotine medications’, ‘Nicotinell’, ‘Nicoderm’, ‘nicotine chewing’, ‘nicotine pastilles’, ‘nicotine delivery system’, ‘Nicorette’, ‘medicinal nicotine’, ‘nicotine tablet’, ‘over-the-counter nicotine’, ‘OTC nicotine’, ‘nicotine orally’, ‘nicotine intranasal’, ‘nicotine polacrilex’, ‘nicotine sprays’, ‘nicotine sublingual’, ‘nicotine lozenges’ and ‘nicotine inhalers’. These keywords were used to search titles. Although people reported that using electronic cigarette (EC) may reduce cigarette smoking consumption and help quit smoking [42, 43], EC was not included as an avenue of nicotine replacement. Little evidence is known about EC, and relatively little research on this issue has been performed [44–46]. In a recent systematic review about EC that health care providers are recommended to be aware that EC devises are of unknown safety and of unsure help as an effective smoking-cessation strategy [47].

Most of the time, NRT term is used as a general term in many different types of sciences. So, to avoid this confusion, the following keywords: ‘smoking’ and ‘tobacco’ were entered as ‘article title, abstract, keywords’. The bibliometric analysis is determined by looking at all past years up to December 31, 2016 and all citations analysis were accomplished on December 22, 2017 to avoid any updating on the database [14]. Research output in the years 2017 was expelled from further analysis. Furthermore, erratum documents were excluded from analysis.

The NRT research productivity was analyzed as previous similar bibliometric studies [2, 14, 24–27] by examining the relative growth rate, citations patterns, collaborative measures, the most prolific institutions and journals. The indicators of bibliometric evaluation including countries, journals, cited articles, and institutions were transformed to the rank order by using the standard competition ranking (SCR) (i.e. “1224” ranking) as in previous similar bibliometric studies [33, 34, 48–50]. Only the ten top-ranked of the measurements were taken into account. In addition, the impact factor (IF) for the ten top-ranked journals was derived from 2016 Journal Citation Reports (JCR). Furthermore, the h-index (also known as Hirsch index) was used as qualitative measure to assess the scientific research performance in the field of NRT for the ten top-ranked countries. The *h*-index was introduced by J. Hirsch in 2005 [51] and is defined as follows: “A certain country has an *h*-index, if it has at least h publications for which it has received at least h citations”.

### Statistical analysis

All extracted data from Scopus were analyzed using Statistical Package for Social Sciences software (SPSS, version 15.0). Frequency, percentage, median, and interquartile range (IQR: i.e. Q1–Q3, lower quartile–upper quartile) were considered. Research activity for the top 10 countries were adjusted to the population size (publications per 1 million inhabitants) and the gross domestic product (GDP) value (publications per $1 billion USD of GDP) retrieved from the online databases of the World Bank [52], and data associated with prevalence of tobacco smoking retrieved from the online databases of the World Health Organization [53]. Pearson’s correlation coefficient test was used to assess the correlations between number of publications and the countries’ population and economies indicators or prevalence of tobacco smoking. *P* < 0.05 was considered significant. In addition, VOSviewer software version 1.6.6 [54] was used to build bibliometric diagrams for visualization the co-occurrence network of terms extracted from the title or abstract of the articles, and to visualize the collaboration network between countries.

## Results

**Fig. 1:**
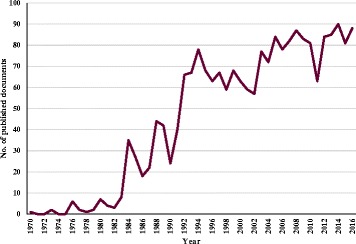
The total articles included in a bibliometric analysis of worldwide publications related to nicotine replacement therapy for tobacco cessation from 1970 to 2015

Using the methodology presented above, 2138 documents related to the use of NRT in tobacco cessation were retrieved. Among them, 1533 (71.7%) were original journal articles, distantly followed by note (171, 8.0%), letters to the editor (160, 7.5%), review articles (149, 7.0%), and other document types such as editorials (125, 4.8%). The yearly production trend increased steadily from 1970 to 2016, and showed a clearer trend to increase in the last years (Fig. 1). The first article related to NRT for tobacco cessation in Scopus was published by Jarvik et al. in *Clinical Pharmacology & Therapeutics* in 1970 [55]. Most documents published in the field of NRT were in English (*n* = 1961; 91.7%), followed by German (*n* = 55; 2.6%), French (*n* = 31; 1.4%) and Spanish language documents (*n* = 30; 1.4%).

**Fig. 2:**
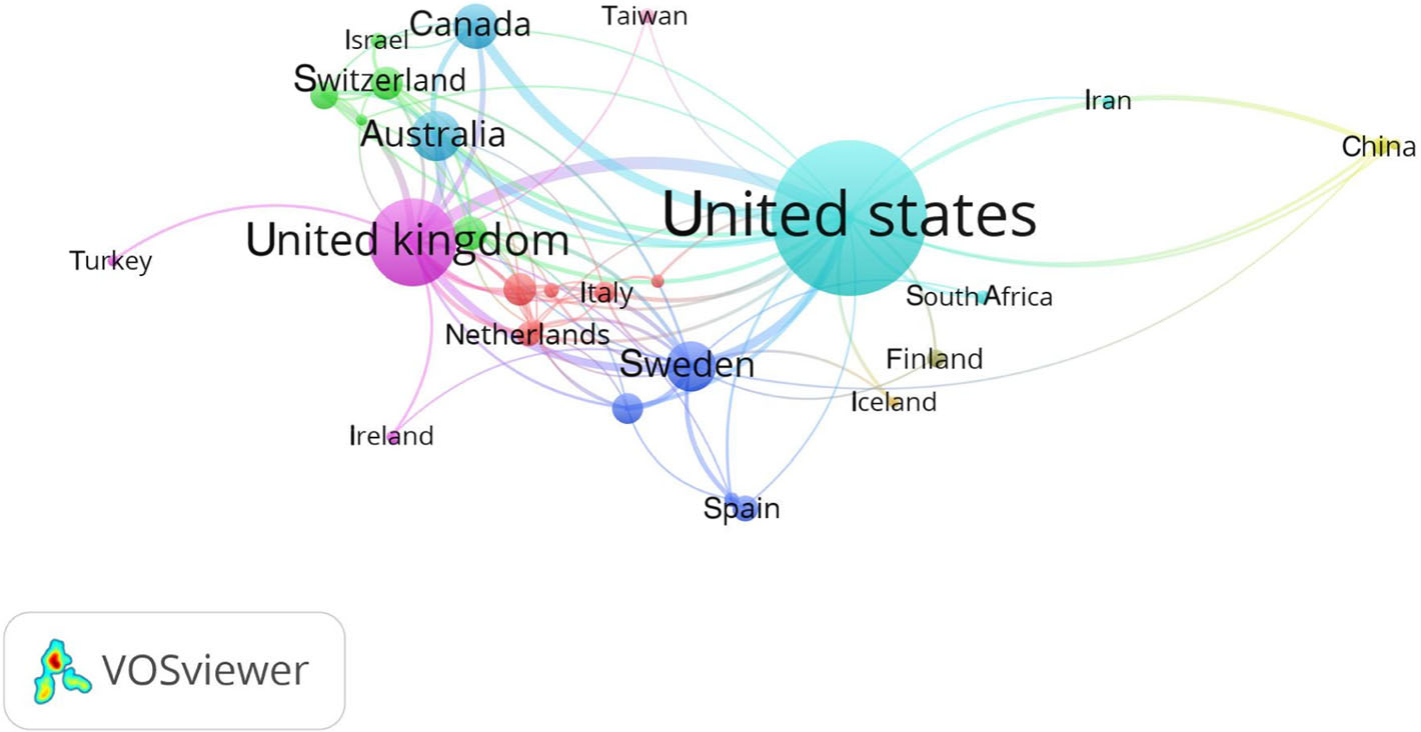
Network visualization map of country co-authorships. Of the 56 countries, 35 had at least five publications; the largest set of connected countries consists of 35 countries in 10 clusters

**Fig. 3:**
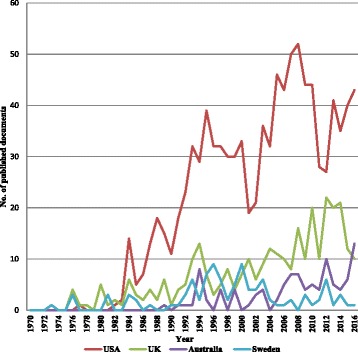
Annual number of publications for top 4 productive countries during 1970–2015

The 2138 documents on NRT research were published by research groups in 56 countries/territories. Figure 2 shows the collaboration network of countries who had at least five publications. The thickness of lines indicates the size of collaboration and the size of circles signifies the number of publications of the country. In addition, the USA produces the most international collaborative documents, with involvement from 32 countries, followed by 21 countries for the UK and 19 for Sweden. The top 10 countries published 1809 documents, accounting for 84.6% of the total number of publications. The USA has the most number of published articles accounted to 986, followed by the UK (312 publications) and then Australia (102 publications), and Sweden (102 publications); (Table 1). No data related to NRT were published from 156 (73.6%). The publication tendency of the most active 4 countries among the first 10 countries in the field of NRT was revealed in Fig. 3. After adjusting for population size, Sweden, New Zealand and Denmark become the most prolific country by achieving the highest number of publications per population size in million inhabitants (10.303, 7.249, and 7.155, respectively). Additionally, after adjusting for the socio-economic parameters, New Zealand, Denmark, and the UK become the most prolific country by achieving the highest number of publications per *GDP (current US$)* in billion (0.184, 0.134, and 0.118, respectively); (Table 1). No significant correlation was found between the country population size or 2016 GDP values and the number of publications of the top-10 most prolific countries in the field of NRT (*r* = − 0.156, *P* = 0.664; and *r* = − 0.173, *P* = 0.632, respectively). Furthermore, there is no correlation between prevalence of tobacco smoking and number of publications of the top-10 most prolific countries in the field of NRT (*r* = − 0.235, *P* = 0.514).

**Table 1:** The top 10 ranking of the most productive countries in publishing the largest number of articles associated with nicotine replacement therapy for tobacco cessation during the period from 1970 to 2016

SCR^a^	Countries	Articles (%)	*h*-index	Publications per population size in million inhabitants	Publications per *GDP (current US$)* in billion	Prevalence of tobacco smoking^b^	Number of Publications per 100,000 smokers	Collaborations with foreign countries	Number (%)^c^ of documents with international authors
1st	United States	986 (46.1)	92	3.051	0.053	17.2	1.77	32	127 (12.9)
2nd	United Kingdom	312 (14.6)	57	4.753	0.118	19.2	2.48	21	98 (31.4)
3rd	Australia	102 (4.8)	25	4.227	0.085	14.9	2.84	8	35 (34.3)
3rd	Sweden	102 (4.8)	41	10.303	0.198	20.6	5.00	19	56 (54.9)
5th	Canada	85 (4.0)	23	2.342	0.056	14.9	1.57	6	33 (38.8)
6th	France	51 (2.4)	16	0.762	0.021	27.6	0.28	11	12 (23.5)
7th	Germany	49 (2.3)	13	0.593	0.014	30.3	0.20	11	14 (28.6)
8th	Switzerland	47 (2.2)	21	5.615	0.070	23.2	2.42	9	17 (36.2)
9th	Denmark	41 (1.9)	22	7.155	0.134	17.0	4.21	8	12 (29.3)
10th	New Zealand	34 (1.6)	15	7.249	0.184	16.3	4.45	6	18 (52.9)

*SCR* Standard Competition Ranking

^a^Equal countries have the same ranking number, and then a gap is left in the ranking numbers

^b^Age-standardized prevalence of current tobacco smoking among people aged 15 years and older [53]

^c^Percentage of documents with international authors from the total number of documents for each country

The total number of citations for the published articles related to the use of NRT in tobacco cessation was 57,333, with a median of citations (interquartile range) nine (9–31) which achieved 26.8 citations per document. Moreover, the *h*-index for the published articles related to the use of NRT in tobacco cessation was 113 (113 articles had been achieved citations at least 113 times), and the highest h of an individual was 92 for the USA, followed by 57 for the UK.

The top 10 most productive journals are summarized in Table 2. *Nicotine and Tobacco Research* ranked first and published 143 (6.69%) articles on NRT. *Addiction* published the second most articles (77, 3.60%), followed by *Psychopharmacology* (61, 2.85%), and Addictive Behaviors (54, 2.53%). Furthermore, all the top 10 most productive journals had an official IF more than 2. It was shown that the USA was the most prolific country for the top 10 most productive journals (Table 2). Medicine was the most researched area, achieved by 1760 (82.3%) articles, followed by pharmacology, toxicology and pharmaceutics with 485 (22.7%) articles, and psychology with 167 (7.8%) articles.

**Table 2:** The top10 ranking of journals in which articles associated with nicotine replacement therapy for tobacco cessation were published worldwide

SCR^a^	Journal	country of journal’s origin	Frequency (%)	IF^b^	Most productive country (No. of documents-%)
1st	*Nicotine and Tobacco Research*	UK	143 (6.69)	4.609	USA (99-69.2)
2nd	*Addiction*	UK	77 (3.60)	5.789	USA (34-44.2)
3rd	*Psychopharmacology*	Germany	61 (2.85)	3.308	USA (34-55.7)
4th	*Addictive Behaviors*	UK	54 (2.53)	2.944	USA (36-66.7)
4th	*Tobacco Control*	UK	54 (2.53)	5.469	USA (35-64.8)
6th	*British Medical Journal*	UK	37 (1.73)	20.785	UK (31-83.8)
7th	*JAMA - Journal of the American Medical Association*	USA	33 (1.54)	44.405	USA (29-87.9)
8th	*Drug and Alcohol Dependence*	Netherlands	28 (1.31)	3.222	USA (23-82.1)
9th	*Annals of Internal Medicine*	USA	27 (1.26)	17.135	USA (18-66.7)
10th	*Journal of Consulting and Clinical Psychology*	USA	25 (1.17)	4.593	USA (24-96.0)
10th	*Preventive Medicine*	USA	25 (1.17)	3.434	USA(18-72.0)

*SCR* Standard Competition Ranking, *IF* impact factor

^a^Equal journals have the same ranking number, and then a gap is left in the ranking numbers

^b^The impact factor was reported according to Institute for Scientific Information (ISI) journal citation reports (JCR) 2016

**Fig. 4:**
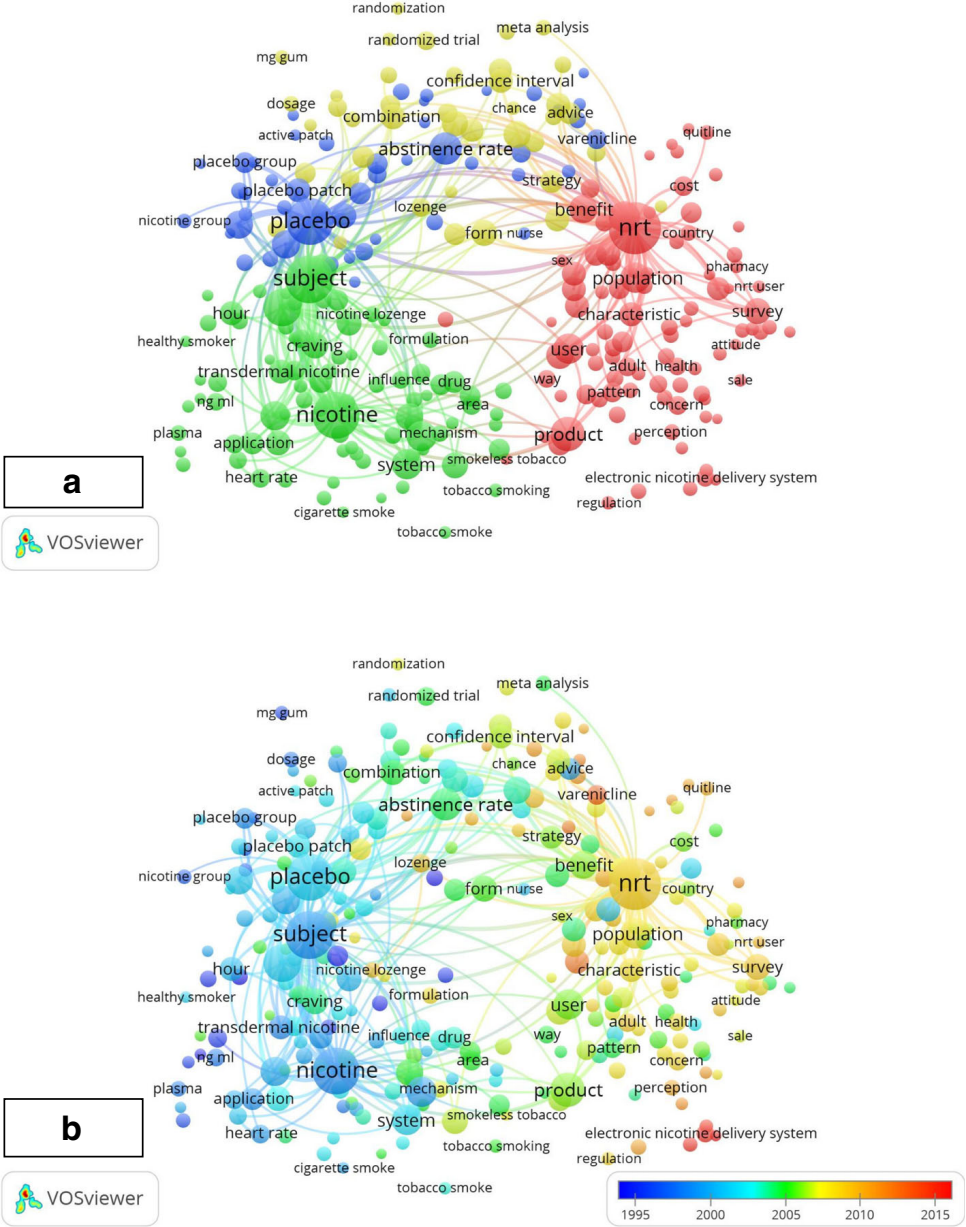
Mapping of terms in the research on nicotine replacement therapy (NRT). **a** shows the co-occurrence network of terms that extracted from the title or abstract of at least 10 articles. Colors represent groups of terms that are relatively strongly linked to each other. The size of a term signifies the number of publications related to NRT in which the term was appeared, and the distance between two terms represents an estimated indication of the relatedness of these terms. **b** represents the earlier (blue color) or later (red color) years when the term appeared

The co-occurrence network of terms that extracted from the title or abstract of at least in 10 articles are presented in Fig. 4a. Overall, 445 of the 23,444 terms meet the threshold of co-occurrence and the top 60% with the highest relevance, that is, 267 terms are set out in the figure and were classified into the 4 clusters according to different colors. The blue cluster mainly represents randomized clinical trials in which NRT in different dosage forms was compared to placebo. The yellow one include terms mainly grouping topics related to related to randomized clinical trials in which NRT in different dosage forms was compared vs. varenicline vs. bupropion vs. Combination. while the red cluster normally implement terms related to population survey studies regarding attitudes, practices, perceptions, and barriers in tobacco cessation. Moreover, the green cluster includes side effect of NRT and pharmacokinetic terms as well. Terms were color coded by VOSviewer based on the average time they founded in the 2138 related publications (Fig. 4b). Figure 3b represented the earlier (blue color) or later (red color) years when the term appeared. Before 2010, in the early stage of NRT research, the main popular topics were related to clinical trials of NRT. The latest trends showed that the main popular topics were related to population survey studies regarding NRT.

**Fig. 5:**
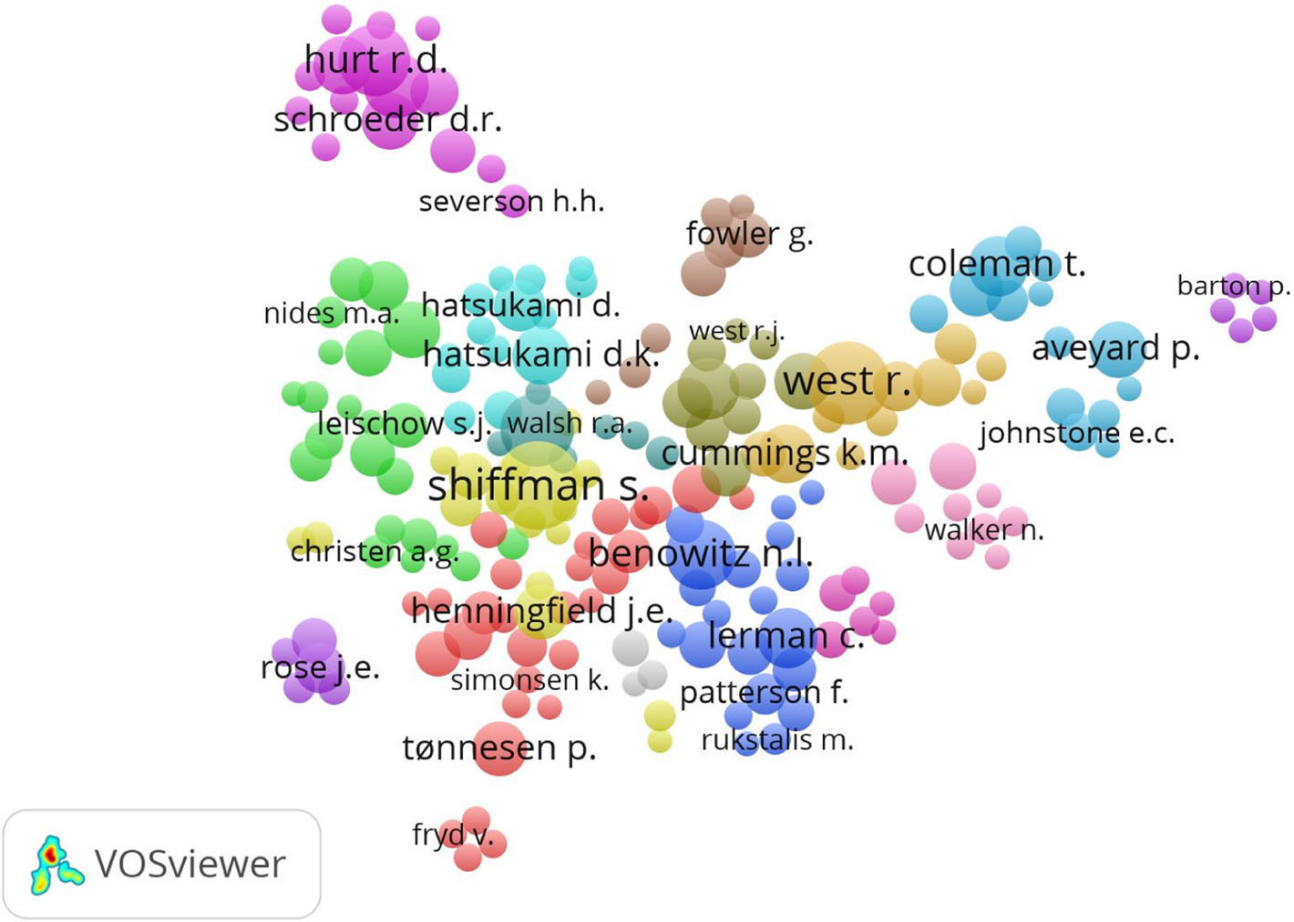
Network visualization map of the authors. Of the 4969 authors, 205 had at least five publications; the largest set of connected authors consists of 195 authors in 16 clusters

Table 3 shows the articles with high citations from 1970 to 2016. Number of citations ranged from 1348 to 325 [6, 39, 56–63]. Table 4 illustrates the top 10 prolific institutions ranked by the numbers of publications. The most productive institution was the VA Medical Center, USA (3.18%), followed by King’s College London, UK (3.13%), and University of Pittsburgh, USA (3.04%). For authors who had the most publications in the field of NRT (Table 5), Shiffman, S ranked the first (59 publications), followed by Benowitz, NL (54 publications), and West, R (53 publications). The cooperation between authors was elucidated in a network map (Fig. 5).

**Table 3:** The top 10 ranking of cited articles worldwide associated with the use of nicotine replacement therapy for tobacco cessation in Scopus

SCR^a^	Authors with year of publication	Title	Source title	Cited by
1st	Jorenby et al., [59]	“A controlled trial of sustained-release bupropion, a nicotine patch, or both for smoking cessation”	*New England Journal of Medicine*	1348
2nd	Fiore et al., [58]	“The effectiveness of the nicotine patch for smoking cessation: A meta-analysis”	*Journal of the American Medical Association*	547
3rd	Stead et al., [6]	“Nicotine replacement therapy for smoking cessation”	*Cochrane Database of Systematic Reviews*	536
4th	Silagy et al., [63]	“Meta-analysis on efficacy of nicotine replacement therapies in smoking cessation”	*Lancet*	400
5th	Benowitz and Gourlay, [56]	“Cardiovascular toxicity of nicotine: Implications for nicotine replacement therapy”	*Journal of the American College of Cardiology*	381
5th	Pullan et al., [62]	“Transdermal nicotine for active ulcerative colitis”	*New England Journal of Medicine*	381
7th	Joseph et al., [60]	“The safety of transdermal nicotine as an aid to smoking cessation in patients with cardiac disease”	*New England Journal of Medicine*	379
8th	Benowitz et al., [57]	“Nicotine absorption and cardiovascular effects with smokeless tobacco use: Comparison with cigarettes and nicotine gum”	*Clinical Pharmacology and Therapeutics*	371
9th	Kenford et al., [61]	“Predicting smoking cessation: Who will quit with and without the nicotine patch”	*Journal of the American Medical Association*	334
10th	Stead et al. [39]	“Nicotine replacement therapy for smoking cessation”	*Cochrane Database of Systematic Reviews*	325

*SCR* Standard Competition Ranking

^a^Equal articles have the same ranking number, and then a gap is left in the ranking numbers

**Table 4:** Top 10 ranking of highly productive institutions that most frequently published articles associated with the use of NRT in tobacco cessation worldwide

SCR^a^	Institutions	No. of documents (%)
1st	VA Medical Center, USA	68 (3.18)
2nd	King’s College London, UK	67 (3.13)
3rd	University of Pittsburgh, USA	65 (3.04)
4th	University of California, San Francisco, USA	58 (2.71)
4th	University of Minnesota Twin Cities, USA	58 (2.71)
6th	University College London, UK	54 (2.53)
7th	Mayo Clinic, USA	51 (2.39)
7th	Pinney Associates, USA	51 (2.39)
9th	University of Vermont, USA	42 (1.96)
10th	University of Nottingham, UK	41 (1.92)

*SCR* Standard Competition Ranking

^a^Equal institutions have the same ranking number, and then a gap is left in the ranking numbers

**Table 5:** Top ten prolific authors who published the most frequently articles associated with the use of NRT in tobacco cessation worldwide

SCR^a^	Author	No. of documents (%)	Affiliation
1st	Shiffman, S.	59 (2.76)	*University of Pittsburgh, Pittsburgh, PA, USA.*
2nd	Benowitz, N.L.	54 (2.53)	*University of California, San Francisco, CA, USA*
3rd	West, R.	53 (2.48)	*University College London, London, UK*
4th	Hatsukami, D.K.	46 (2.15)	*University of Minnesota, Twin Cities. Minneapolis, MN, United States.*
5th	Hughes, J.R.	44 (2.06)	*University of Vermont, Burlington, VT 05401, USA*
6th	Hurt, R.D.	40 (1.87)	*Mayo Clinic, Rochester, MN, USA*
7th	Croghan, I.T.	34 (1.59)	*Mayo Clinic, Rochester, MN, USA*
8th	Tønnesen, P.	30 (1.40)	*Glostrup Hospital, Glostrup, Denmark*
9th	Lerman, C.	28 (1.31)	*University of Pennsylvania, Philadelphia, PA, USA*
10th	Coleman, T.	26 (1.22)	*University of Nottingham Medical School, Queen’s Medical Centre, Nottingham, UK*
10th	Cummings, K.M.	26 (1.22)	*Medical University of South Carolina, 67 President Street, Charleston, SC 29425, USA*

*SCR* Standard Competition Ranking

^a^Equal authors have the same ranking number, and then a gap is left in the ranking numbers

## Discussion

Reducing morbidity and mortality associated with tobacco use involves an understanding of how scientific research related to tobacco use has progressed. Such study is helpful in implementation or developing of an efficient strategies to respond to this progress [25, 64]. To my knowledge, this is a first study that using bibliometric approach analysis to estimate the NRT literature indexed in Scopus database at global level. Bibliometric indicators in the current study illustrated that research output in the field of NRT was ignored in most countries. The current study also adds to the emerging international bibliometric studies in the field of tobacco smoking [2, 14, 16–20], which may open doors to new research on the evaluation of effective smoking-cessation strategies and counselling services.

However, the first publications related to NRT were introduced in the early 1970s, the *Lancet* was published in 1942 paper entitled ‘Tobacco smoking and nicotine’ by Johnston who recognised smoking as one of the most important factors for lung cancer and later he considered one of the first anti-smoking campaigners by suggesting a total ban on smoking [65]. Johnston reported that he had administered a subcutaneous small dose of nicotine solution to 35 volunteers, including himself [65]. He declared that if nicotine injections were abruptly discontinued, craving to tobacco will be arise. He demonstrated that smoking tobacco (i.e. smoking addiction) is actually a means of administer nicotine, similar to smoking opium is essentially a means to administer morphine [65, 66]. Jarvik and colleagues published the first article about the use of orally administered nicotine for tobacco substitutes in 1970 in *Clinical Pharmacology & Therapeutics*. Jarvik et al. found that nicotine administration produces a significant decrease in the average number of cigarettes smoked [55]. Later, Jarvik became the co-inventor of the nicotine patch. Three years later, Ferno and colleagues developed a chewing nicotine gum that released nicotine at a suitable rate when chewed [67]. A preliminary report given at the Second World Conference on Smoking and Health in 1971 in London compared the nicotine-containing chewing gum with a placebo gum in a double blind study [68], and the results were published in 1973 in *Psychopharmacologia* [69].

The USA was the most prolific country, accounting for 46.1% (*n* = 986) of total output. Furthermore, the USA was the most collaborative country in the world and got the central position in the collaborative network. This finding was similar to other recent studies related to waterpipe tobacco smoking and EC [14, 28]. The current research indicates that Australia has published the greatest number of research articles from the Asia–Pacific region, while Sweden has published the majority of research articles from Europe. The 10 most prolific countries to publish articles on NRT consist of numerous nations that will be similar to other scientific output rankings [70]. This research output from these countries is possible due to fact that these are: a) countries with more resources [71], b) countries experiencing high rates of tobacco smoking [53], c) countries with a research and publishing culture in relation to tobacco smoking [72–75], and d) countries that publish most journals in the field of health [76]. In the current study, the performance in research output for every country is different. This study demonstrated that the USA and the UK are the most influential countries in the field of NRT. This activity may be associated with the population size, and socioeconomic of these countries [77]. No study has been found in the literature similar to this point, thus the author was interpreting these results in light of other results. These findings are consistent with data obtained in other previous research which found similar findings [78, 79]. Countries with fast-rising economies, which consequently have more funding and investment for conducting research [25], thus may contribute to increasing number of publications regarding NRT.

In this study, the average citation rate was 26.8 citations per article. This citation was higher than the average citation of papers published in toxicological journals [24, 26, 80–82]. A more recent studies using similar bibliometric indicators found the average citation rate for waterpipe tobacco smoking publications was 13 citations per article, and for EC publications was 6.4 citations per article [14, 28]. This finding indicates that NRT become a hot issue in scientific research.

Another important finding was that was the investigation of publications’ quality. To note, all the top 10 journals in which international articles related to NRT were published carried IFs greater than 2.00 and had significant impact in the field of tobacco smoking. Articles from Sweden amassed high *h*-index. This achievement is due to the fact that Sweden developed the first effective therapies for tobacco dependence. Nicotine chewing gum (i.e. Nicorette) was the first therapeutic preparation aiding in smoking cessation which was developed by Ove Fernö at Leo pharmaceutical company in Sweden and was first approved in Switzerland in 1978 [83], following an idea from Fernö’s colleagues, Stefan Lichtneckert and Claes Lundgren, at the Physiological Institute of Lund University in Sweden in 1967 [67, 83].

It is necessary to take into account a number of limitations as in other previous bibliometric studies [31, 32, 34, 49, 50, 84, 85]. First, the *Scopus* database was used to search for NRT studies. Thus, the contribution of non-*Scopus-*publication may have been underestimated. Second, the chosen key words might not be comprehensive. Therefore, false negative results are a possibility. Third, some articles did not contain NRT and related keywords in the publication titles. Therefore, NRT and related keywords were mentioned inside the text or abstract were not included in this study. Another limitation that this bibliometric analysis results in a significant bias in the sense that the researcher has little or no control over the key problems associated with a lack of production of studies related to NRT.

## Conclusions

The present data reveal a solid mass of research activity on NRT. The USA attained a leading position in global NRT research, with the largest number of independent and international collaborative publications. NRT-based research activities were low or not available in most countries. As NRT research has been thought to be generally useful to humans, more efforts should be taken to further research in this field. The current study provides useful information to researchers and funding societies concerned in the implementation of research strategies to improve NRT research for small economies or to address global health issues related to tobacco control services. Furthermore, the findings of this study demonstrate that use of NRT in tobacco cessation remains a hot issue in scientific research. Moreover, side effects, pharmacokinetic aspects of NRT, survey population studies, and clinical trials have been recognized as the most prominent hotspots in the research related to NRT. Additionally, The results of this study delineate a framework for better understanding the situations of current NRT research and prospective directions of the research in this field which could be applied for managing and prioritizing future research efforts in NRT research.

## Abbreviations

GDP: Gross domestic product
IFs: Impact factors
IQR: Interquartile range
ISI: Institute for Scientific Information
JCR: Journal citation report
NRT: Nicotine replacement therapy
SPSS: Statistical package for social sciences
WHO FCTC: World Health Organization Framework Convention on Tobacco Control
